# Hospital and Institutional News

**Published:** 1912-08-24

**Authors:** 


### HOSPITAL AND INSTITUTIONAL NEWS.

### THE HOSPITAL CONFERENCE IN BIRMINGHAM.

The annual Conference of the British Hospitals Association will be held in Birmingham on September 19 and 20. The opening day will include a reception at the Council House by the Lord Mayor, and the 150 or 200 delegates expected to be present will have the opportunity of visiting several hospitals in the city and district. Mr. Howard Collins, House Governor of the Birmingham General Hospital, is in charge of the local arrangements. At present we will content ourselves with drawing attention to one or two of the papers to be read. Sir William Collins and Mr. J. Danvers Power have promised to contribute—the former a paper on "Hospitals and the State" and the latter one upon "Hospital Management Considered in Relation to Responsibility,"—and, in view of the recently published Report of King Edward's Hospital Fund on the Reform of Out-Patients, many will note with interest a paper on "The Training of Almoners," which Mr. Edgar S. Kemp, Secretary of the Hospital Almoners' Council, will read. Mr. Kemp, our readers will remember, recently wrote an article on this subject in The Hospital. Dr. Nathan Raw will write on "The Insurance Act." All who attended the memorable Conference at Manchester last September, which bore fruitful results in the deputation to the Chancellor on the Insurance Act, will remember how successful and enjoyable it proved, and there seems little doubt that Birmingham this year will prove as attractive and inspiring to all the delegates who are fortunate enough to attend.

### THE NON-COMPLIANT DOCTORS ON THE INSURANCE COMMITTEE.

Fourteen medical members of the Insurance Advisory Committee, whose names we give below, have refused to comply with the British Medical Association's request to resign their posts. The signatories were Dr. Addison, M.P., Sir Clifford Allbutt, Dr. Clement Belcher, Mr. C. J. Bond, Sir John Collie, Mr. Adam Hamilton, Mr. M. St. L. Harford, Mr. Herbert Jones, Dr. Arthur Latham, Dr. H. H. Mills, Sir Shirley Murphy, Dr. George Reid, Dr. John Robertson, and Professor Sims Woodhead. And at the end of last week Dr. C. Addison, Liberal member for Hoxton, wrote a letter to the medical secretary of the Association explaining the grounds of his refusal. He argued that membership of the Advisory Committee was too useful a lever for securing the claims of the profession to be thrown away; that the Association itself had required that medical men should be appointed on the Advisory Committee; and that, while stating its willingness that medical men should work the sanatorium benefit, the Association forbade them to serve on the Provisional Insurance Committees, the almost sole business of which, says Dr. Addison, is to administer this benefit. He concluded his letter by saying, "I will take no part in a boycott of which I am ashamed."

### TWO POINTS OF VIEW.

The fourteen who have declined to resign are taking a step which places them in direct opposition to the British Medical Association. Undoubtedly they make out a fairly good case for themselves—as individuals; that is, they advance certain arguments for disagreeing with the Association, and those arguments, if not unanswerable, at least must be taken seriously. But whether or not these gentlemen conscientiously hold it better that the members of the Advisory Committee should not resign, their action in refusing the request of the Association is unfortunate and may be disastrous. They are opening a serious rift in that new-found solidity of the profession, just when it is most important to preserve an unbroken front. They are setting themselves up in opposition to the considered policy of the vast bulk of the profession. The tactical details of that policy may or may not be the best attainable; but this kind of disloyalty and disunion spells certain defeat. There is yet another aspect of the case which is suggested by a perusal of the list of the fourteen—several of those whose names are the best known are keen politicians of the advanced radical school; one, indeed, is a member of Parliament, commonly credited with aspirations. The idea will inevitably be suggested that these gentlemen are sacrificing their professional brethren to retain the good will of the Chancellor of the Exchequer, or to become placemen.

### A LABOUR MEDICAL CANDIDATE FOR PARLIAMENT.

Within a few hours of the time when this issue reaches our readers, the result of the Parliamentary by-election at East Carmarthenshire will be known, and it will be decided whether the scanty band of medical men in the House of Commons—reduced by the death of Dr. Hillier since the last General Election—is to receive a new recruit or not. For one of the three candidates is a practising medical man, standing in the Labour interest for election; this is Mr. J. H. H. Williams, L.S.A., of Burry Port, Carmarthenshire. It seems very doubtful whether Mr. Williams will be successful in his candidature; but if he should be, the advent of a qualified medical man to the benches of the Labour Party in Parliament might possibly result in greater sympathy there with the efforts of the medical profession to avoid exploitation in connection with the National Insurance Act. It does not appear from medical works of reference that Mr. Williams has taken any conspicuous part in medical politics or in those developments of State medicine which are becoming so increasingly important; but his experience as a general practitioner in a district which is, as a whole, by no means affluent, should enable him to speak authoritatively on problems of the moment which affect especially the average general practitioner. Even apart from this the profession generally is awakening to the importance of being represented in the House of Commons, where previously it has been so conspicuous by its absence, and so disregarded by the other professions from which the ranks of politicians are recruited.

### THE REGISTRATION OF STILL-BIRTHS.

We are glad to see that the Report of the Special Committee of the Royal Statistical Society to inquire into the various systems of registration, on which we commented last week, is attracting attention to the importance of registering still-births. At present, as the inevitable result of non-registration, the causes of still-births are not scientifically tabulated, but the moment it is remembered that one of these causes is syphilis, it will be seen how important it is to know exactly how many still-births are due to this cause. It is, indeed, odd that the immense strides which registration has already made should have neglected a field on the face of it so full of rewards for the statistician and the sociologist. The recent attempts to lessen infantile mortality must remain unnecessarily incomplete so long as the number of still-born is not known, and the causes of still-birth are not investigated. One of the chief values of statistics, indeed, is found to be not merely the tabulation of causes, but the popularising of them among a previously indifferent public. The effect on the public mind of new statistics has often proved quite extraordinary. Once the profession is able to say to parents and mothers one baby out of every three is still-born because, whatever the cause may be, the lesson proves almost unescapable. Mrs. Bulley, in a letter to the newspapers, goes so far as to suggest that "if school health officers would state plainly in their reports what was the cause of the injured eyes of school children things would begin to mend." But it must be remembered that the causes of many diseases are too doubtful at present not to give an offended parent, able to afford this revenge, a reasonable chance of a verdict for libel. And the whole cause of vital statistics would lose by any such attempt at abuse.

### THE SATURDAY FUND AT LEICESTER.

Sir Edward Wood (president) was in the chair at the half-yearly meeting of the Leicester Saturday Hospital Society held at the Royal Infirmary last Saturday. The Committee's Report dealt with the introduction of the National Insurance Act, and mentioned difficulties which had been experienced in the collection of the weekly pence, but the conclusion arrived at was that "the difficulties were but temporary, and that as soon as the workpeople have become accustomed to the new conditions, and when they realise that the Act, comprehensive as it is, has its limitations, there will be no doubt as to the attitude of the working men and women in their support of the Society." Alluding to the new branch of the Society's work—the opening of a convalescent home for female members—the Report stated that the Swithland Home was opened on May 2, when 28 patients were admitted, since which time 130 patients had been received. The total cost of the Home was £16,763. The total number sent to homes during the half-year was: males, 434; females, 277; total 711: an increase of 95 patients on the corresponding six months of 1911. The total expenditure was £1,182 3s. 3d. The Report was adopted on the motion of the Chairman, supported by the Vice-Presidents, Mr. S. A. Gimson and Mr. J. Gipson Clarke, the Mayor of Leicester (Ald. Tollington), and Mr. C. J. Bond, F.R.C.S. Readers of other notes in these columns this week will not fail to note that opinions still differ as to the effect of the Insurance Act on working-men's subscriptions, and experience varies in different parts of the country.

### LADY DOCTORS' WORK AT FEZ.

The Ladies' Medical Mission at Fez has received a visit from General Lyautey, the Resident General of the French Republic, and, according to the *Times* correspondent, received from him a donation of £20 as a mark of his appreciation of its work. A number of Moorish women receive medical aid from the ladies, chief of whom is Miss Mellett, whose work among them is well known. General Lyautey made a speech in which he not merely told the patients how grateful they ought to be for the "charity of the British ladies," but emphasised the fact that his own difficult work was facilitated by the mission. The work of Miss Mellett and her friends is often one not without danger, but they are so much respected in Fez that, in the opinion of those who should be able to judge, they are regarded as practically free from all risk of ill-treatment.

### THE AMERICAN HOSPITAL CONFERENCE.

The programme of the fourteenth Annual Meeting of the American Hospital Association to be held at Detroit, Michigan, from September 24 to 27, includes the following papers: "Social Service at the Massachusetts General Hospital," by Miss Ida M. Cannon; "The Economic Features and Feeding of Hospital Employees and Patients," by Dr. H. T. Summersgill; "Economy in the Operating-Room," by Mr. Asa Bacon; "A Contribution to the Problem of Convalescence," by Dr. Fred. Brush; "The Use of Salvarsan ('606') in Hospitals," by Dr. Ross; "The Cost of Infectious Diseases," by Prof. James W. Glover; "The Relation of the General and Special Hospitals in the Care of the Insane," by Dr. Chas. K. Clarke; "The Present-Day Obligations of Hospital Trustees," by Mr. Richard E. Borden; "Hospital Management, Especially the Division and Responsibility of Labour," by Mr. J. R. Coddington; "The Hospital Interne," by Dr. Reuben Peterson; "Hospitals and their Duty in Relation to the Prevention of Disease," by Dr. Chas. P. Emerson; "The Hospital Laundry," by Dr. Winford H. Smith; "Hospital Organisation, with Special Reference to that of the Detroit General Hospital," by Dr. W. F. Metcalf; "The Equipment of a Hospital Kitchen," by Mr. A. A. Wilson; "Some Social Aspects of the Hospital," by Rabbi Leo M. Franklin. In addition, there will be the Question Box and the Round-table Conference for workers in the smaller hospitals, as at previous meetings. A Trustees' Session will also be held. An advance list of papers is not generally very exciting reading, but we think all will admit that there should be life as well as comprehensiveness in the wide scope of the papers, the titles of which we have quoted. Some of the subjects chosen are original beyond the common.

### THE SECRETARY AS EDITOR.

Our valued contemporary, *The Hospital Gazette*, has changed its editor, owing to the resignation of Mr. Stewart Johnson having taken effect on his election as President of the Incorporated Association of Hospital Officers. The post of assistant editor has also been created, and it is interesting to note that the two posts have been filled by contributors to our own columns. The new editor, Mr. George Watts, Secretary of the City of London Hospital for Diseases of the Chest, was the author of a valuable illustrated article on "A New Form of Shelter for a Number of Patients Simultaneously," which appeared in these columns a few months ago; while the new assistant editor, Mr. F. P. Carroll, Assistant Secretary at Charing Cross Hospital, still more recently contributed an article on "Water Softening Appliances," a technical subject of hardly less interest than difficulty. On these grounds alone our readers will join with us in congratulating our contemporary on the new members of its staff, who have already proved themselves interesting writers on institutional technics. Mr. Stewart Johnson, who became editor towards the end of 1909, has maintained the monthly issues at a commendably high level of interest.

### TWO SECRETARIES HONOURED.

To mark their appreciation of the efforts by which the annual church parade of the Ibstock Friendly Societies produced over £21 for the Leicester Royal Infirmary and the annual fête of the Broughton Astley Hospital Society the sum of £26, the Board of Governors have asked the committees of the respective societies to nominate one of their number to a Life Governorship of this Royal institution. It is a cause of satisfaction that the nominees for this honour are the Hon. Secretaries of the respective movements, Mr. J. W. Hopkins and Mr. W. F. Dear, upon whom much of the organisation of the parades has rested.

### SIR WILLIAM OSLER'S ADVICE.

In an exceedingly interesting letter on the subject of the tuberculosis campaign, Sir William Osler argues for making the dispensary the centre of the fighting line, for the institution of instruction in diagnosis, and for attaching to every general hospital with a medical school a tuberculosis department. "All are agreed," Sir William states, "that the fighting line will centre about the dispensary. No provision exists for the training of their officers, who should have special instruction in methods of work, and particularly in diagnosis. The appointments will be well paid, and the public has the right to demand well-trained men. It should be a duty of the general staff to arrange suitable courses at the dispensaries and special hospitals. In London it would be easy to arrange an attractive three months' programme, utilising the Brompton Hospital, the existing tuberculosis dispensaries, and the Lister Institute. In Edinburgh Dr. Philip and a few of the younger men at the Royal Infirmary could arrange courses for Scotland. The diagnosis of tuberculosis is often very difficult, mistakes are common, mistakes are going to cost much money, and it will pay the public to demand that the men in charge of the dispensaries are thoroughly equipped for the work. To make provision for the training of this officers' corps should be the first duty of the tuberculosis subdepartments of the Local Government Board." Sir William then instances the tuberculosis dispensary in connection with the Johns Hopkins Hospital as one which "forms an important part of a great medical school, through which every student as a matter of routine passes as a clinical clerk. If for no other purpose than this every general hospital with a medical school should have its tuberculosis department." Do not, he concludes in effect, spend large sums on sanatoria until the establishment and co-ordination of dispensaries throw light as to where and to what extent they are needed. A more fruitful letter has seldom appeared in the *Times*, and we shall be glad to publish any criticisms of Sir William Osler's proposals which our readers may feel inclined to offer.

### THE NEW DISPENSER AT THE WEST END HOSPITAL.

The vacancy at the West End Hospital for Diseases of the Nervous System, which was caused by the death of the dispenser under tragic circumstances (reported in The Hospital, August 10), has been filled. The committee have appointed Mr. Robert Fouracre, M.P.S., F.S.M.C., to the post. Mr. Fouracre, who has been on the dispensing staff at the London Hospital for several years, passed his qualifying examination in 1903, and took the higher diploma of pharmaceutical chemist quite recently. In addition to these distinctions, he has studied optics and is a Fellow of the Spectaclemakers Company. He has also served in the dispensaries at the Metropolitan and Charing Cross Hospitals.

### THE NEW HOSPITAL AT FOWEY.

Last week Mrs. Treffry, of Place, laid the foundation-stone of the new hospital at Fowey, which is on the Place estate and has been given by its owner. Mr. C. E. Treffry has promised to supply all the local stone which may be needed for building purposes, and the hospital itself will be virtually a single-storeyed building, though the nurses will be accommodated on a dwarf upper storey. The wards are to be three in number: a men's ward with four beds, a special ward for private patients, and a women's ward with three beds. A theatre is to be provided, and a verandah will run along the whole south front of the building. The cost is expected to be £2,000, of which one-half is already in hand, and the architect is Mr. O. W. Parkes Lees, a resident in the neighbourhood. As soon as the ceremonial laying had been performed, Mr. C. E. Treffry laid on the stone a cheque for £50 from Mrs. Treffry and himself, together with the deeds for the conveyance of the land. The President of the new institution, Dr. Rashleigh, then proposed the health of the Prince of Wales, and Canon Purcell, the chairman, who has been connected with the institution for forty-six years (the hospital was founded in 1860), made an eloquent appeal for funds. Canon Purcell, indeed, is the sole surviving trustee. Mr. J. H. Hannan, the honorary secretary, who has explained that the new hospital is not an extension but a change of habitat, stated that the old hospital would be sold, and that £900 more was needed, but that the six parishes served by the hospital should be able without much difficulty to raise that sum. A bazaar was opened later by Mrs. Rashleigh, who remarked that it had been organised by the ladies of Fowey, who were determined not to be outdone by the gentlemen. There can be no doubt, in short, that the new institution had a very enthusiastic "send off," and the way in which the hospital has grown to meet the needs of the district augurs well for its latest development.

### MR. MAYO ROBSON RETIRES.

The announcement has been made public that Mr. Mayo Robson, C.V.O., F.R.C.S., is retiring from active practice. Mr. Robson's long and distinguished career fully entitles him to take a rest, which we trust he will long enjoy. As an example of difficulties surmounted by dint of hard work and dogged determination to succeed, Mr. Robson's professional life-story will always be an incentive to young men in the profession who have ambitions. In a recently published volume of papers by the staff of St. Mary's Hospital, Rochester, U.S.A., one of the celebrated Mayo brothers speaks very highly of Mr. Robson's operative technique in an article detailing his impressions of various British surgeons whose work he witnessed when visiting England a few years ago.

### HOSPITAL POLICY IN CARDIFF.

King Edward VII.'s Hospital, Cardiff, is an example of the institutions which the Insurance Act is beginning adversely to affect. Mrs. Henry Lewis, Greenmeadow, and Mr. Isaac Samuel, J.P., both alluded last week to the risk of opening more beds with a decreasing income, though the waiting list now numbers 900. General Lee, who was in the chair at the August meeting, then read a letter which had been sent to subscribers, from which we quote: "The Board of Management point out to all subscribers, and especially to the bodies of workmen who have so loyally supported the hospital for many years, that the provisions of the Act will not supersede the necessity for hospital treatment. It is confidently hoped that some relief will ultimately be afforded to the pressure on the Out-patient Department, but the provisions of the Act will not avail for the treatment of accidents or for patients requiring serious operations, as for cancer, appendicitis, strangulated hernia, etc. Indeed, it is apparent, upon a visit to the wards, that scarcely any of our in-patients could be treated independently of the hospital. The demand on our resources remains as constant as ever, no fewer than 914 patients at present requiring admission, whilst 50 of our new beds remain idle for want of funds. Indeed, at this crisis in the history of the nation's health it is more urgent than ever to place in commission every available hospital bed, as a national system of medical treatment may well reveal conditions hitherto outside the province of any medical man or any medical authority." The letter, which is signed by Mr. Leonard D. Rea, secretary and general superintendent, concludes with a postscript effectively compressing the whole by saying "no medical benefit is provided by the Act for women (who are not personally insured) and children, and these number the majority of patients treated at the hospital."

### STOCKPORT INFIRMARY AND THE INSURANCE ACT.

Some disquiet has been caused in Stockport by the announcement at the monthly meeting of the infirmary that a local firm and their workpeople have decided to discontinue their subscriptions to the institution. The Secretary, Major Tyler, had received a previous intimation to this effect, and had written asking the firm to reconsider its decision. The result was a letter which he read, that contained the following passages: "Profits at the present day will not allow of unlimited subscriptions, and the expense incurred under the Insurance Act is such a serious item that we have perforce to economise in some direction." That this attitude is shared by the employees seems evident from another quotation, which ran: "Our employees say that their pockets will not stand a compulsory deduction on account of the Health Insurance (which many of them have previously been getting much more economically than under the present Act), and at the same time keep up voluntary contributions to the infirmary." We are bound to say that we do not feel too much sympathy with the firm in question. As is well known by hospital secretaries, the subscriptions of employers, often not too generous in amount, are in the nature of insurance, and the extent of treatment for their workpeople which they expect from the hospital as a right is often, if worked out, far more than that to which their subscription entitles them. A firm sends a cheque of £100, say, to the local hospital, and claims at once a sort of lien on the hospital for all cases which may arise. Such a subscription is too often merely a very inexpensive investment for the employers, from which they expect not merely valuable services, but the kudos that comes from public support of charity. How sincere that support really is may be judged by the readiness with which excuses are used for its withdrawal.

### A MILLIONAIRE'S DYSPEPSIA.

It is, perhaps, worth recording the gratitude shown by Mr. James Buchanan Brady, a New York millionaire, to the St. John's Hospital, Baltimore, where some months ago he went as a dyspeptic, and, after prolonged treatment, was operated upon and lately discharged cured. Upon realising, by the practical experiment of indulging as a gourmand in two banquets within three days without dyspepsia, that his former trouble had left him, he informed the hospital authorities of his desire to present £45,000 to its funds. We understand the money will be devoted to the ward in which stomach and kidney troubles are treated, though the most extravagant equipment of one ward could hardly absorb so large a sum.

### MIDWIFERY FEES AND LAMBETH INFIRMARY.

The doctors in North Lambeth evidently have misunderstood to whom they are to apply for payment when summoned on the requisition of a midwife to a case where the persons are too poor to pay a doctor's fee. Consequently, there has been a great increase in the calls on the services of the medical staff at Lambeth Infirmary. Indeed, the number of cases where they have been called in on relieving officers' orders has more than doubled since June 1909, when the Guardians decided to discontinue the payment of a fee of 10s. to the medical officers. The Board at that time gave directions that midwives in North Lambeth should, in cases of necessity, obtain the services of any qualified medical practitioner in the district except their own medical officers, but they have either not acted on the instructions, or the medical practitioners in the district have not thought fit to take the cases, in spite of the fact that their fees would be paid by the Guardians. The difficulty is that if the Guardians incur no expense by allowing their own medical staff to attend these cases they cannot reclaim any sum from the patient's husband, a fact which is likely to lead to serious abuse. In the meantime the doctors in North Lambeth are again to be notified that the Board will pay their fees in midwifery cases of this nature.

### A JOURNALIST'S HOSPITAL BEQUESTS.

The public bequests of the late Mr. J. E. Taylor, of the *Manchester Guardian*, have become payable owing to the recent death of his widow. Manchester, of course, which received much from his generosity during his lifetime, including several Turner water-colours, is well remembered. Victoria University receives £20,000 and the Royal Infirmary £1,000. The largest hospital bequest, however, appears to be £5,000, which is to be devoted to the London Temperance Hospital; thus London rather than Manchester charities may, perhaps, claim his chief affection so far as hospitals are concerned. This is all the more surprising, as in every other respect Mr. Taylor has left his money so markedly to his own city.

### "WHEN WE DEAD AWAKEN."

Of recent years more than one hospital authority has given to the Cheltenham General Hospital the benefit of its advice and criticism, apparently not quite without effect, as the local residents are at last beginning to take up the question of the hospital's management and finance themselves. The *Cheltenham Onlooker* has opened a series of "critical articles dealing with the present parlous state" of the institution. The first of these was an open letter intended, apparently, for Colonel Croker-King, the president, who was held responsible for certain delays and bad management alleged in connection with the enlargement of the hospital, which was inaugurated in February 1910. The opening of the wards is noticed for the fact that no use was made of it to raise money, and that subscribers only were asked. The Colonel is also accused of enforcing red-tape restrictions on the admission of patients, and at the end a broad hint is given to him to retire in favour of a "young, vigorous, tactful, capable man." The worst fault in the institution, however, is not mentioned; that is the nurses' quarters, which were described in The Hospital's Report on the institution (Jan. 15, 1910) as "the very worst to be found anywhere in the south of England." Did this Report produce any effect? Not a bit, save to give to Mr. Sydney Holland the opportunity of remarking after a personal visit two years later that they still merited that description. The financial position is causing great anxiety, and it is interesting to note that The Hospital urged what a false policy it was to reduce the private staff of nurses: "To be safe financially, voluntary hospitals in the present day must meet the requirements of all classes of the population resident in the district which they serve." The Cheltenham institution has neglected perhaps the most important section of the local residents by curtailing its private staff. Is it to be wondered at, therefore, that an increasing indifference has been shown? The late hon. treasurer, Mr. C. L. Grundy, has written in reply vindicating the president, and saying that if any blame is to be attached every member of the house committee must share it. Public indifference to a hospital is always the result of an unfortunate policy on the part of its board of management. It has so consistently rejected suggestions in the past, however, that the local paper's belated effort must not inspire too much confidence of success. Indifference to a hospital is a bad habit which a board that has once allowed it to spread will find it very hard to remove. A recent anonymous gift of £500 is, we hope, the sign of better things.

### WOMEN AS SCHOOL MEDICAL OFFICERS.

The work of medical inspection of school children, as has often been remarked in these columns, demands peculiar gifts, if not a special training, and the school clinic staff is one which demands, though it does not invariably receive, very careful selection. For this work, on occasion, women doctors have proved to have an aptitude, and many will not have failed to notice that the successful candidates for the school medical officers to the London County Council contain women among them. One of the most recent was Miss Mary Gertrude Edis, M.D., who previously had acted as medical officer for the inspection of school children at Stockport. But whatever the sex of the school officer, it is important that he or she should have a much more intimate knowledge of children's diseases than is possessed by the ordinary run of general practitioners, or is sometimes even held desirable by them.

### PRESENTATION TO A MATRON AT MEXBORO'.

A presentation was made last week to Miss Berry, Matron of the Montagu Hospital, Mexboro'; she has been Matron there since October 1903, and is about to be married. The presentation, which consisted of a handsome case of cutlery and a cheque, was made by Councillor John Clayton (chairman of the board). Futher presentations were made by the Ladies' Committee, who gave a grandfather's clock; by the hon. medical staff, who gave a canteen of table silver; and by the nursing staff, whose present was a case of silver and a drawing-room lamp. Miss Berry is succeeded by Miss Wesley, who has been sister for several years.

### STATE MEDICAL SERVICE PROPAGANDA.

The various and disconnected talk with which all have been long familiar as to the desirability of forming a State Medical Service crystallised a few weeks ago into "The State Medical Service Association," when Professor Benjamin Moore presided. Its main object is described as being "to advocate amongst the medical profession the advisability of a State Service, to make clear its advantages to the public, and to promote its adoption by Parliament." It is thus seen that the Association is to undertake the "education" of professional opinion, and it will have produced a useful result if, by a direct referendum, or by the subtler tests of opinion which a well-organised association can generally obtain, it makes clear exactly what attitude the generality of the profession takes up. Hitherto the advocacy of a State Medical Service has generally been undertaken by those who advocate State activity in many other directions, and we do not fail to note the presence of a Socialist or two among the early members of the new Society. The views of Professor Benjamin Moore, whose "Dawn of the Health Age" was noticed on its appearance in these columns, are well known, and, provided that the Association produces not merely propaganda but a measure of professional opinion on this matter, many who are hostile or indifferent to its aim will thank it for a useful piece of work.

### THE POWER OF JOURNALISM.

Never has the power of journalism been more directly proved than by the recent exposure in *Truth* of the Putumayo atrocities. That exposure, in the face of great opposing interests, was a courageous and a really great piece of journalism—the sort of editing, and we can give it no higher praise, that would have delighted and warmed the heart of Henry Labouchere. Mr. R. A. Bennett, his successor in the editorship of *Truth*, has added another to its laurels; and to him is directly due that action which has been taken by the Government. It would be a graceful and well-deserved recognition of his public spirit in exposing the Putumayo horrors if the Government, whose politics he shares, included his name in the next honours list. Hospital workers, who know how much the cause of true charity has gained from the constant exposure of those who abuse it, alike from interest and humanity will welcome any such distinction, which is due both to the man and to the profession of journalism.

### THE ACTIVITY OF THE WOMAN PHARMACIST.

Miss C. Minnie Fox, M.P.S., pharmacist to the Royal South Hants and Southampton Hospital, has been contributing poetry to the local press, and the activity of lady pharmacists is so marked that the desirability of women representatives on the Council of the Pharmaceutical Society has recently been discussed even in a lay paper. The journal was promptly informed that two candidates would be before the electors in May next. This would be direct representation with a vengeance, as the Council of twenty-one members presumably represents the 16,000 or so registered pharmacists, and it is a bold calculation that would calculate one-tenth or one-twentieth of this number to be women.

### HOSPITAL DISPENSERS AND THEIR CRITICS.

Last week we had occasion to criticise some statements concerning hospital training for pharmacists. *The Pharmaceutical Journal*, in its last issue, referred to the same subject, but in a fairer manner than did Mr. Saville Peck. "In recent years," said our contemporary, "no branch of pharmacy has made more rapid strides than the pharmacy practised in hospitals, and some hospital pharmacists now take apprentices. This experience, whilst being perhaps the best attainable in practical pharmacy, leaves the pupil without much business training, and all the routine of shop work has to be learnt after apprenticeship." This is no insurmountable task or we could not explain the large number of hospital dispensers who become successful business men; whilst there are many who spend two or three evenings weekly in a retail pharmacy and find themselves at home. However, our point is that the pharmacy of the hospital or dispensary is decidedly professional pharmacy, and we are glad that the journal of the Pharmaceutical Society recognises this, although it was disparaged by one of their examiners.

### THIS WEEK'S DRUG MARKET.

Business in the Drug market is extremely dull, few articles attracting any attention. Of changes in value few of any note have taken place. Opium is still in an uncertain position; bromides, iodides, and mercurials are unaltered in price. The firm position of quinine is well maintained. There is little demand for cod-liver oil, but the price is not likely to become much lower than it is at present. Cocaine is tending lower in value. The price of atropine has been slightly advanced. The high price of ergot of rye is maintained.

### TO OUR READERS.

Contributions are specially invited from any of our readers to these columns. They should deal with topical subjects and news. They must be authenticated for the information of the Editor only. The minimum payment if published is 5s. There is no hard-and-fast rule as to space, but notes of about twenty lines in length are preferred.

